# Quality of care is what we make of it: a qualitative study of managers’ perspectives on quality of care in high-performing nursing homes

**DOI:** 10.1186/s12913-021-07113-9

**Published:** 2021-10-13

**Authors:** Brigitte Lalude Asante, Franziska Zúñiga, Lauriane Favez

**Affiliations:** Institute of Nursing Science, University of Basel, Bernoullistrasse 28, 4056 Basel, Switzerland

**Keywords:** Nursing care, Leadership, Quality of Health Care, Nursing Homes, Qualitative research

## Abstract

**Background:**

Leadership has a vital role regarding quality of care in nursing homes. However, few studies have explored upper-level managers’ views on how to assure that residents receive high quality of care. Therefore, this study’s aim was to examine how managers of top-quality nursing homes define, develop and maintain high-quality of care.

**Method:**

We used interpretive description, an inductive, qualitative approach. Our research included 13 semi-structured interviews with 19 managers. We analyzed their input using reflexive thematic analysis, which is an iterative approach.

**Results:**

Quality development and maintenance are cyclic processes. Managers in high-performing nursing homes lead with high commitment towards a person-centred quality of care, creating appropriate working conditions and continuously co-creating a vision and the realization of quality of care together with employees.

**Conclusions:**

This study confirms that, in high-performing nursing homes, a person-centered approach—one where both residents and employees are at the center—is essential for quality development and maintenance. The most effective managers exemplify “person centeredness”: they lead by example and promote quality-focused working conditions. Such strategies motivate employees to provide person-centered care. As this means focusing on residents’ needs, it results in high care quality.

## Introduction

Improved standards of living and health care have dramatically increased life expectancy in many countries [1]. The number of older adults receiving care in long-term-care facilities such as nursing homes is increasing [2]. Helping this vulnerable group maintain their health and quality of life while retaining as much autonomy as possible demands high quality care in nursing homes [2]. Yet, factors such as rising number of residents with complex care needs, high staff turnover rates leading to discontinuity of care, organizational process changes or scarce funding often make it difficult to provide nursing home residents safe, high-quality care [2–5].

Faced with comparable challenges in similar contexts (e.g., concerning legislation or financing models), some nursing homes deal with issues much more successfully than others [6]. Even where the overall quality is satisfactory, differences in care quality are clearly visible between facilities [7–9]. In Switzerland, even though the majority of nursing home residents rate their overall quality of care (92.8 %) and life (71.3 %) as *good* or *very good* [5, 10], recently developed Swiss quality indicators showed a wide range of quality between nursing homes [7].

The need to deliver high quality care places heavy demands on nursing home managers, who need to fill residents’ and their families’ growing needs with scarce available resources [2, 11]. Differences in quality of care result partly from nursing home managers’ relative competencies and abilities to address such challenges [12]. These managers must assure resident quality and safety, provide strategic direction, and establish necessary structures and processes—all while encouraging and guiding staff development to achieve targeted outcomes and strategic goals [12–14].

Nursing home managers refer to two different roles: nursing home directors (NHDs) and directors of nursing (DONs). And while both of these roles are essential to nursing homes’ organizational effectiveness, they differ greatly in their assignments [14]. NHDs typically manage their facilities’ daily operations. This role calls for leadership, but also involves managerial, commercial, strategic and legal responsibilities [11, 14]. DONs often serve as a second-tier manager who reports directly to the NHD. In addition to a nursing degree, DONs need to demonstrate clinical competencies and effective leadership skills [15]. Most Swiss nursing homes’ organizational models place DONs in co-leadership positions alongside NHDs; others assign both roles to the NHD.

Nursing home managers often influence how challenges are faced within their facilities and decide how safety standards and quality of care are developed, achieved and maintained [14, 16–19]. This means their leadership approaches and behaviors (e.g., regarding communication or supervision), as well as their roles, capabilities and even years of experience in their position can all affect outcomes regarding both residents (e.g., satisfaction with care, safety, physical restraint use) and staff (e.g., job satisfaction, turnover) [16, 19–21].

Ample evidence suggests that nursing home managers can have a close relationship with quality of care. However, few researchers have examined the details of that relationship: there is little evidence on just how managers’ leadership characteristics influence the quality of care provided in their care contexts [6, 14, 19]. To bridge this gap, a Swedish study explored the perspectives of high-achieving managers. Specifically the researchers explored how the most effective managers promoted person-centered care in their nursing homes [22]. Based on the values of respect, individuality, autonomy and empathy, person-centered care is associated with improvements in resident outcomes including well-being [23–25]. In examining this relationship, previous researchers have described essential aspects of quality of care development [22], but have not fully explored how nursing quality can be promoted and maintained in nursing home contexts. With this study, we aim to explore first how nursing home managers from high-performing Swiss nursing homes define quality of care, then how they develop and maintain it.

## Method

### Design

We used Thorne’s interpretive description methodology, an inductive, qualitative approach developed for applied research [26]. By guiding data description and interpretation, Thorne’s methodology facilitates the understanding of clinically relevant phenomena. Applying it to each studied manager’s description of how they promote quality of care resulted in useful knowledge for nursing practice.

### Setting and study participants

This work was embedded in the Swiss Nursing Homes Human Resources Project 2018 (SHURP 2018). Using a convenience sample of 118 nursing homes in the German and French speaking regions of Switzerland, the SHURP study examined the relationships between organizational characteristics and quality of care [27]. To be included in the study, nursing homes had to be recognized as such by their cantonal authorities. Among the participating facilities, 86 had already updated their routine needs assessment instruments to allow collection of data on the six newly introduced Swiss quality indicators. These covered four domains: physical restraint, weight loss, polypharmacy and pain. The definitions of the six Swiss national quality indicators for nursing homes can be found in Table 1 [7].

 Quality of care was gauged via the six national quality indicators. For this study’s purposes, each home was assigned one rank for each quality indicator. We calculated the final ranking based on the sum of the ranks across all six indicators. We invited managers of nursing homes ranked among the top 30 % to participate in this sub-study. We invited participants for interviews if they were currently working in an institutional-level leadership position, either as an NHD (administrative leadership), or as a DON (clinical leadership). To be eligible, participants had to fulfill three criteria: (1) they had been in their current nursing home position for at least one year prior to SHURP 2018; (2) they worked a minimum employment percentage of 60 % in a German-speaking Swiss nursing home; and (3) they spoke German. We restricted the study to the German-speaking part since only 1 French-speaking NH had provided data on quality indicators. At facilities where the NHD and DON were separate positions, some facility managers collaborated so closely with their DONs that they asked for and were granted joint interviews. To minimize desirability bias the researchers did not establish a relationship with any of the participants before the beginning of the study.

**Table 1:** Definitions of the national quality indicators for nursing homes in Switzerland

Quality Indicators	Definition
Physical restraint	(1) Percentage of residents with daily fixation of the trunk or with seating that prevented the resident from rising in the last 7 days (2) Percentage of residents with daily use of bedrails or other devices on all open sides of the bed that did not allow the resident to leave the bed independently in the last 7 days
Weight loss	(3) Percentage of residents with weight loss of 5 % or more in the last 30 days or of 10 % or more in the last 180 days
Pain	(4) Self-reported pain: Percentage of residents with daily moderate or higher pain intensity or residents with nondaily very strong pain intensity in the last 7 days (5) Observed pain: Percentage of residents who showed daily moderate or higher pain intensity or residents who showed nondaily very strong pain intensity in the last 7 days
Polypharmacy	(6) Percentage of residents who took 9 or more active ingredients in the last 7 days

### Data collection

 We initially contacted managers in 19 nursing homes by e-mail to participate in the study. Six did not fit the inclusion criteria, mostly because of management-level changes. In total, we recruited 19 study participants—12 NHDs and 7 DONs—from 13 nursing homes. Those who agreed to participate were contacted by the first author to make an interview appointment. The first author, who works as a geriatric nurse in a university hospital, conducted eight individual and five group interviews with 2–3 persons each. Interviews were semi-structured, guideline-based and face-to-face; they took place between September 2019 and January 2020. All started with the question “what defines high quality of care from your point of view?” The interview guide contained open-ended questions on communication, leadership styles, activities carried out to ensure quality, dealing with challenges, as well as general questions to explore managers’ views regarding quality of care. The interview guide was developed based on a prior literature review on how quality of care can be improved and on discussion among the research team members. Some key questions are found in Table 2. Other topics mentioned by the managers during the interviews were further explored through in-depth questions. All participants also completed a short sociodemographic questionnaire.

 Conducted in participants’ nursing homes, the interviews lasted 43–77 min. They were audio-recorded, then transcribed into standard German. Redundant data with recurring statements and common themes were achieved through the 13 interviews, indicating acceptable data saturation. Field notes were written immediately after the interviews and used during data analysis. All participants were informed at the beginning of the interview both that their data were confidential and that they could withdraw at any time. All signed a declaration of informed consent. The study received an ethical waiver from the Northwest and Central Swiss ethics committee (Req-2019-00608).

**Table 2:** Key questionnaire items to elicit examples

Topic	Questions
General	What defines high quality of care from your point of view?
Leadership	How would you describe your role’s contribution to the quality of care in your nursing home? How is quality promoted and maintained in your nursing home?
Communication	What defines “good” communication from your point of view? How do you shape communication in your nursing home?
Challenges	How do you address challenges that may arise when dealing with nursing staff in everyday life?

### Data analysis

To interpret the interview data coherently and convincingly, the authors used Braun et al.‘s (2019) iterative method of reflective thematic analysis—a six-step approach to identifying, analyzing and highlighting issues [28]. Performing analyses in parallel with data collection allowed us to evaluate topics that emerged from the earlier interviews and add them to the guide. We then explored those issues further in later interviews. As suggested by Braun et al., the first author familiarized herself with the data by transcribing the interview input, then reading the transcripts several times. With each iteration, she first made, then revised interpretations.

The analysis also included coding, continuously interpretating and comparing all interviews to identify patterns. Topics were generated and displayed using thematic maps. The MAXQDA 2020 software program [29] was used for data analysis. The coding, analysis and theme building were discussed regularly with the second and last authors. A working group for qualitative research provided quality assurance by discussing the analytical steps.

## Results

**Fig. 1:**
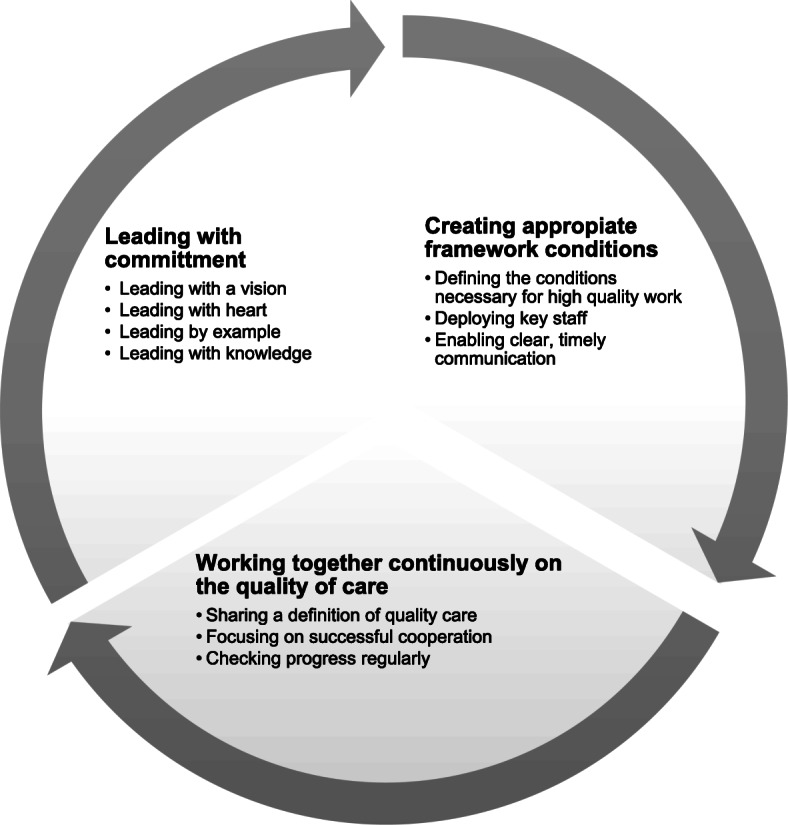
Results: main topics and sub-topics

The study received input from 19 participants (NHDs and/or DONs) in 13 interviews. Information on the participants can be found in Table 3. Our analysis showed that, from these managers’ shared perspective, attaining high-quality nursing care is about leading with commitment, creating appropriate framework conditions and working together continuously on quality of care (see Fig. 1).

**Table 3:** Participants’ sociodemographic information (*n* = 19)

Participants’ sociodemographic data	n (%)
Gender Female	16 (84.2)
Age in years, mean (range)	50 (37–64)
Current function Nursing home director (NHD) Director of nursing (DON)	12 (63) 7 (37)
Length of service in current function in years, mean (range)	9.8 (1.5–25)
Length of service in nursing home in years, mean (range)	9.1 (1–25)
Occupational field Nurse Architect and activation specialist Primary teacher	17 (90) 1 (5) 1 (5)
Training/further training areas Management-specific Care-specific Both	13 (70) 1 (5) 5 (25)

### Leading with commitment

Ensuring that residents received high quality of care required purposeful, *fully committed managers* with *vision, heart, and knowledge*, who led their staff *by example.* All the nursing home managers emphasized the value of a clear *vision* of quality of care. Most either started with an overarching vision of quality or generated one with the cooperation of all stakeholders. One person described it as follows:

It helps that I have an idea of what level of quality I want to stand up for. [7]

From these managers’ point of view, their passion for and enjoyment of their work in the nursing home influenced the quality of care. It was important to them that both they and their staff members not only recognized but were also intrinsically motivated to fulfil the purpose of their work. One interviewee made it clear that they led with *heart*:

I myself also have a certain passion for the job. I do this myself with conviction [3].

These managers were conscious both of setting an *example* by leading honestly, reliably, transparently, with perseverance and adaptability, and of developing the ability to reflect on their actions. As they lived these qualities personally, they asked no less of their employees. The same applied to other professional and/or personal qualities: they demanded nothing from their staff members that they themselves could not (or would not be able to) deliver. As one DON summed it up:

It is important that I live up to what I expect from my employees. And that is something very central for me in my leadership style [2].

These managers recognized that advancing their nursing homes’ quality of care demanded both leadership acumen and professional expertise. Their recognition that *knowledge* and skills enabled them to coach and empower employees drove them to overcome personal knowledge deficits either via personal study or via further/advanced training. Nursing home managers were able to maintain pleasant working relationships through their leadership styles and their ability to identify employee needs, which they considered as sympathetically as possible when creating framework conditions.

### Creating framework conditions

Nursing home managers believed that the underlying purpose of their job was to create suitable conditions for high quality of care. This involved *defining the conditions necessary for high quality work*, *enabling clear, timely communication* and *deploying key staff.* Regarding high quality of care, all interviewees agreed it was necessary to lay out clear prerequisites for those involved in care delivery and to *define the conditions necessary for high quality work*, that is, factors that regulate the entire care institution’s structures and processes. Many also noted that transparent, systematic management was essential:

A…systematic approach, this is very central. This helps not only to have an orientation, but also to give people the feeling that they are not lost in the quality discussion [1].

 Their strategies to make or keep their systems transparent included working together to create and adapt quality documents such as guidelines, concepts and instructions. These had to be not only feasible but fully understandable. One described the experience in terms of changing one’s point of view:

I am trying to move up from the [staff at the] base. So today I look much more at: What does the base need? With which concepts can [they] work so that it is simply clear (emphasized) for every woman, every man? [12].

For many respondents, this change of perspective was vital to the creation of framework conditions that allowed them to identify their employees’ needs. To explore staff`s perspectives on given working conditions, identify gaps in coverage, discuss challenges and plan measures to overcome them, all managers *enabled clear and timely communication* through regular inter- and intra-disciplinary exchanges and an open communication culture. Nursing home managers supported this culture partly by being present in everyday workplace life and partly by signaling their accessibility via visits to the units. Of course, it was not enough simply to be physically present: they also had to contribute to a comfortable atmosphere. One interviewee focused on awareness of others:

We also attach importance to being aware of each other. Both among staff and with relatives or residents, this gives a basic message that ‘One is welcome, one is noticed.’ [7].

All nursing home managers agreed that mistakes are unavoidable but can be learning opportunities. They considered it extremely important for employees to communicate errors: in addition to minimizing any damage, this allowed them to address both individual and systemic deficits. To identify and resolve difficulties, managers *deployed key staff*, i.e., people who had a decisive influence on residents’ quality of care. Some of these people worked alongside nursing professionals in the residential areas to deal with critical situations. They were deployed both to bring in useful empirical knowledge and to translate that knowledge into practice. Managers distinguished between key clinical and key managerial staff. Key clinical staff are nursing specialists with extended knowledge and expertise. They work on-site to guide nursing teams’ practices.

Most of the participating nursing homes had nurse specialists in place, but with various backgrounds. They ranged from registered nurses with some in-depth continuous education in geriatrics or palliative care to advanced practice nurses with master’s-level education. One NHD described why she considered one of her nurse experts particularly useful:

She actually has the professional responsibility in the care groups. She is an important part of the care team, including at the residents’ bedsides. She can manage complex situations together with the team [7].

Key managerial staff, on the other hand, were characterized by their management expertise. As team leaders who also had organizational responsibilities, they had a comprehensive view of what was happening in their units and promoted staff and team development.

### Working together continuously on quality of care

All respondents described the improvement of quality of care as a complex, continuous process that depended on input from all relevant stakeholders, especially residents, relatives and staff. They considered this process cyclical: it required a joint *definition of care quality* linked to active and conscious work towards quality, as well as a strong *focus on successful cooperation* and *regular progress checks*.

Initially, it was necessary to formulate a uniform *definition of nursing care quality*. However, as individual perceptions of nursing care quality sometimes differed, some managers found it important to develop a shared understanding of quality together with all staff involved with care in the nursing home. While defining care quality was sometimes perceived as difficult, all those interviewed focused on holistic, individual care and support for the residents. One participant connected this quite concisely with the principle of person-centered care:

For me, a high or good quality of care is when a treatment…is adapted to the needs of the residents—respecting [the resident’s] wishes and their perspective and responding to them as well as possible while placing them in the center [10].

This type of statement illustrates how high-performing managers consider person-centeredness crucial to quality of care. Other management staff expressed similar perceptions regarding quality of life and person-centeredness in their individual definitions of quality of care. Since the improvement of quality of care is an active process, there are two important implications: first, that the care aspect is extremely important, and second, that both managers and employees act attentively and responsibly in their daily practice. Participants considered quality of care not simply a theoretical ideal, but a specific goal of each day’s work that should be noticeable and visible to residents. One manager described quality building and maintenance as a continuous responsibility:

To maintain quality of care, to keep it moving, that is like a permanent plate that rotates. … For me, this is really a standing order in management. For me it has always been so important: there is no quality unless we make it [4].

The dynamics of quality production are there for all to see. Leaders must ensure that the persons involved cooperate well despite this complex and dynamic process to improve quality of care. However, quality of care never results solely from management efforts: it demands *successful cooperation* and positive synergies throughout their institution(s). Successful managers promote quality by ensuring that, in addition to appropriate professional competencies, each recruit demonstrates highly developed social skills, a sense of responsibility, self-reflection and adaptability.

 Our participants also spoke of continuously strengthening these skills in all staff members. They achieved this via a combination of personal participation and a willingness to share or even transfer responsibility. As a major factor of a positive work environment, this mix influenced first their staff’s work motivation, then the quality of their nursing care. Our participants recognized that each new level of autonomy helped their care staff to feel valued, which motivated them to do still better work. As one manager said:

I call for participation. So, I want them to think along with me—that they themselves, [when] problems arise, think first about how they can solve them together [1].

As one major management responsibility is to ensure that their entire organization is always developing, these continuous, conscious efforts towards improvement of the care quality must also be actively checked at both the system and the individual level. At the system level, our participants initiated periodic reviews to evaluate processes and structures. At the individual level, checks involved having those involved reflect on their actions. These evaluations also depended on residents’ and their relatives’ evaluations of the care and support provided.

## Discussion

As nursing home managers take on various roles, they assume varying ranges of responsibilities. Based on our interviews, they contribute to quality production in three main ways: (1) by leading with commitment; (2) by creating framework conditions favorable to high-quality care; and (3) by collaborating actively and continuously with their staffs to improve the quality of care provided. Because these types of contributions interact in ways that allow managers to review and adjust the relevant framework conditions, these managers ensure that the quality of nursing care can be continuously improved.

In the literature, researchers commonly describe nursing homes as *complex adaptive systems*: they are learning healthcare systems. As agents of these systems, nursing home managers and staff must continuously learn and reorganize themselves in the face of dynamic, interdependent elements and constraints. Relations between the various agents influence resident outcomes. Therefore, quality-oriented managers facilitate both continuous learning and continuous action [16, 30]. That is, they encourage their staff to teach, learn from and collaborate with one another. The effects of these inter-agent relationships were visible in our results.

As quality production in nursing homes consists of numerous interrelated elements as well as dynamic relationships between the participants, their systems are complex. And as many of the needs they fill are unpredictable, those same systems need to be adaptable. To consistently oversee, understand and harmonize the many dynamic processes that shape life and work in such complex systems, high-performing nursing homes’ managers not only have a wide range of abilities and skills, but also tremendous flexibility.

To varying degrees, our interviewees’ personal attitudes, behaviors, characteristics, competencies and commitment levels reflected the ones of leaders managing complex adaptive healthcare system [31]. They also generally shared several character traits. For example, all described themselves as committed, honest, transparent, reliable and self-reflecting. Their ability to reflect on themselves as well as on current processes and structures allowed them to act and adapt to situations appropriately. As noted above, this capacity to adapt is crucial in nursing home. All interviewees also reported leading with a clear vision of quality, i.e., they were aware of exactly what they were working for and why.

Before the strategic goals of the nursing home could be implemented, structural and process-related prerequisites for good nursing care quality had to be defined and in place. The literature confirms that high-quality nursing home care begins with a clear vision in the mind of the manager [30]. Aligning the organization to achieve effective outcomes and realizing that vision requires adjusting structures, functions, and responsibilities [32, 33]—all while adapting to constantly-changing challenges.

For managers, translating their visions into action requires communicating them in ways that allow staff members to fully understand both the short- and long-term goals of their work. Achieving high quality in complex adaptive systems demands open communication and the use of formal and informal information channels. By enhancing employees’ perceptions that their opinions and input are valued, open communication from management fosters open communication from and between staff members [6, 30].

Moreover, we suspect that nursing home managers who influence quality the most are those who not only shape their facilities’ culture but embody it. Intrinsically motivated, fully committed to quality, they lead by example. This is reflected in the literature, which describes how the most effective nursing home managers focus their abilities and skills on fostering leadership, communication, cultural design and cooperation with their staff [6, 12].

Our study results also highlight another of the managers’ central responsibilities: promoting a work culture that recognizes the value of collaboration and person-centeredness. Person-centered care promotes healthy relationships between all participants. This begins by recognizing each resident’s personality and right to self-determination, and treating them with respect and empathy [25].

Within high-performing complex adaptive systems focus on connectivity and person-centeredness is placed. Sullivan et al. [6] highlight person-centeredness as a critical factor in nursing homes. In their 2018 study comparing high- and low-performing nursing homes based on both clinical and person-centered care outcomes, they noted five domains in which the higher- and lower-rated facilities differed: leadership support, organizational culture, teamwork and communication, resident-centered care recognition (including awards), and resident-centered care training [6]. These themes were also prominent in our interviews. From our interviewees’ general perspective, quality meant meeting their residents’ individual needs in a person-centered manner, allowing them to experience a good quality of life.

Our participants also stated that the members of their care teams used various skills and abilities for person-centered care. Recognizing that using all staff members’ knowledge and expertise improves the residents’ daily care [22], they also stressed the principle that staff members who were aware of their importance regarding quality production were more likely to act accordingly. Participants regarded diverse skills as an enriching factor. To encourage it, they acknowledged and supported their staff members in diversifying their skill sets. They noted that when they valued, appreciated and promoted commitment in individuals, one result was that they fostered healthy working synergies. Another was that, as direct attention to care team members’ needs and strengths allowed them to experience person-centeredness for themselves, it led to increases in person-centered patient care.

In practice, then, to maintain their staff members’ motivation—and ultimately quality— managers needed to value and develop those employees’ knowledge and skills. Likewise, as noted by Forbes-Thompson et al., for the purpose of quality production, focusing on the needs not only of residents but also of care teams is crucial [30]. So, while managers practiced person-centeredness regarding their residents, they did the same for their staff. They acknowledged their staff members’ needs and contributions by providing a range of opportunities to share their opinions and then considering and acting on their input.

A 2020 study observed that successful nursing home managers led with a high degree of person-centeredness [22]. While that study asked nursing home staff to evaluate their facilities’ person-centeredness subjectively, we evaluated the targets of person-centeredness based on clinical quality indicators. Despite differences in personal focus, participating managers reported very similar behaviors in both studies. This similarity likely reflects the principle that, like quality, person-centeredness is promoted by managers. The most successful nursing home managers have a personal understanding of person-centeredness and its implementation. They also support employees by exemplifying person-centric attitudes and creating a trustful working environment [22].

A 2020 review by Siegel and Young [14] highlights major research gaps regarding administrative and clinical leadership in nursing homes. The current study’s results are a first step towards a better understanding of that topic. For example, Siegel and Young highlighted the shortage of knowledge available on how NHDs and DONs work together [14]; our findings indicated that, in the most successful care homes, they normally collaborate closely, commonly regarding themselves as partners in producing quality of care. Because most DONs are regularly present on the wards and interact directly with staff members, they are able to bridge many gaps between the wards and the NHD. In their everyday work, DONs use their insights to convey current issues or topics discussed in the wards (e.g., equipment or materials needed) to the NHD. Armed with this information, the NHD deals with the matter of balancing resource requests with budgeting constraints. Siegel and Young also emphasize the need for more research regarding role perception and leaders’ effectiveness, particularly regarding how to build capacity in others and empower staff.

Our results show that NHDs and DONs—mostly DONs—build close relationship with the staff, create opportunities for open communication and generally work to provide room for staffs’ interests, abilities and capabilities to unfold. For managers to perform their roles effectively, they need to rely on the information their DONs can gather, as well as on their own clinical and leadership knowledge [34, 35]. The combination of these resources enables them e.g., to coach employees in ways that will build their capacities [17, 22]. Our participants specifically acknowledged this need, describing how they shared their know-how to support employees’ development [17, 22]. Considering managers’ abilities to interact closely with staff members, to identify and respond to their needs and to have a personal understanding of person-centeredness, our study participants seem to adapt a transformational leadership style to lead successfully. Transformational leaders are not only known for their social competences, i.e., as they are able to foster relationships, motivation and job satisfaction among staff but, like our study participants, they also perceive management as a collaborative and dynamic process [36, 37].

Finally, Siegel and Young’s review shows how little is known about nursing home managers’ preparedness for their roles—especially considering that no further education is mandatory for ward nurses to become DONs. All of our study participants—both NHDs and DONs—had completed further education, mostly in management. In terms of preparatory training, minimum standards are clearly necessary. All study participants also stated that they prepared for their roles as quality promoters by being ready and willing to acquire the necessary knowledge on their own in order to achieve their set goals.

There is no doubt that managers influence the quality of care offered in their care homes. However, the mechanisms of quality production remain largely unexplored. This study offers insights into how high-performing nursing homes’ managers use their leadership capacity to deal with challenges, lead their staff efficiently and ensure high-quality clinical and person-centered care, while managing the uncertainty of their facilities’ complex adaptive systems. Further research is recommended to identify best practices in long-term care management, and to build much-needed leadership capacity (through recruitment, education and training) for long-term care sector managers.

A detailed, in-depth understanding of the mechanisms and competences that successful nursing home managers mobilize to achieve and improve quality of care—what they do and how they do it—implies a possibility to enhance all such managers’ competencies. This study’s participating NHDs and DONs all considered it essential to foster each staff member’s sense of responsibility to develop and enhance not only their residents’ quality of care but their overall quality of life.

## Limitations

Certain limitations should be noted concerning this study. First, participants were aware of their nursing home’s own ranking before the start of the interview. This might have limited their reliability. That is, high rankings may have both motivated them to participate and biased their statements. Second, this study used quality indicator measures as a proxy for quality of care. However, these indicators measure only what they are designed to measure, i.e., they do not measure broader concepts of care such as residents’ quality of life. Still, they do provide information about basic security measurements (e.g., physical restraint use, pain management) known to affect residents’ quality of life.

## Conclusion

This study supports the hypothesis that, in nursing homes, both resident- and employee-centeredness are essential factors of quality production. Our sample of nursing home managers provided mainly homogeneous data that showed similar basic strategies for quality production. While enabling the development of working conditions conducive to high-quality care, employee-centricity contributes considerably to how staff members perceive and carry out their tasks. Therefore, care quality development depends largely both on the presence of appropriate framework conditions and on cooperation through person-centered attitudes shared by managers and staff. This ongoing shift in healthcare culture towards person-centeredness requires not only full commitment on the part of managers but also adequate financial and human resources. Most importantly, our participants confirmed the power of role modelling to motivate their nursing homes’ staff to focus on their residents as individuals and to provide them with high-quality of care.

## Data Availability

The datasets used and analyzed during the current study are available from the corresponding author on reasonable request.

## Abbreviations

DON: Director of Nursing
NHD: Nursing Home Director
SHURP: Swiss Nursing Homes Human Resources Project
